# A Spatial Analysis of COVID-19 in African Countries: Evaluating the Effects of Socio-Economic Vulnerabilities and Neighbouring

**DOI:** 10.3390/ijerph182010783

**Published:** 2021-10-14

**Authors:** Samuel O. M. Manda, Timotheus Darikwa, Tshifhiwa Nkwenika, Robert Bergquist

**Affiliations:** 1 Biostatistics Research Unit, South Africa Medical Research Council, Pretoria 0001, South Africa; Tshifhiwa.Nkwenika@mrc.ac.za; 2 Department of Statistics, University of Pretoria, Pretoria 0028, South Africa; 3 Department of Statistics and Operations Research, University of Limpopo, Sovenga 0727, South Africa; timotheus.darikwa@ul.ac.za; 4 Ingerod, SE-454 94 Brastad, Sweden; robert.bergquist@outlook.com

**Keywords:** coronavirus (COVID-19) pandemic, country-level disparities, spatial regression analysis, Sub-Saharan Africa

## Abstract

The ongoing highly contagious coronavirus disease 2019 (COVID-19) pandemic, which started in Wuhan, China, in December 2019, has now become a global public health problem. Using publicly available data from the COVID-19 data repository of Our World in Data, we aimed to investigate the influences of spatial socio-economic vulnerabilities and neighbourliness on the COVID-19 burden in African countries. We analyzed the first wave (January–September 2020) and second wave (October 2020 to May 2021) of the COVID-19 pandemic using spatial statistics regression models. As of 31 May 2021, there was a total of 4,748,948 confirmed COVID-19 cases, with an average, median, and range per country of 101,041, 26,963, and 2191 to 1,665,617, respectively. We found that COVID-19 prevalence in an Africa country was highly dependent on those of neighbouring Africa countries as well as its economic wealth, transparency, and proportion of the population aged 65 or older (*p*-value < 0.05). Our finding regarding the high COVID-19 burden in countries with better transparency and higher economic wealth is surprising and counterintuitive. We believe this is a reflection on the differences in COVID-19 testing capacity, which is mostly higher in more developed countries, or data modification by less transparent governments. Country-wide integrated COVID suppression strategies such as limiting human mobility from more urbanized to less urbanized countries, as well as an understanding of a county’s social-economic characteristics, could prepare a country to promptly and effectively respond to future outbreaks of highly contagious viral infections such as COVID-19.

## 1. Introduction

The coronavirus disease 2019 (COVID-19), which is caused by a novel beta-coronavirus named severe acute respiratory syndrome coronavirus 2 (SARS-CoV-2), was first reported in the city of Wuhan, Hubei province, China [1,2,3]. It has now spread to all countries and continents and has become the worst and most devasting pandemic in recent times [4,5]. The pandemic has resulted in disastrous and dramatic adverse effects on all human populations worldwide. There is growing empirical evidence suggesting T substantial inter-country variations in the levels of COVID-19 risk and impacts due to varied economies, health systems, social strengths, and vulnerabilities, [6,7,8].

As of 31 May 2021, the number of confirmed cases and deaths in Africa and globally had risen to more than 4.8 million and 130,000, corresponding to case fatality rate (CRF) = 2.70%; and 170.3 million, 3.7 million, and CRF = 2.15%, respectively. Globally, the five countries with the highest COVID-19 burden as of 31 May, 2021 were the United States of America (USA) with 33,554,274 cases, 602,092 deaths, and CRF = deaths 1.7%; India with 29,935,211, 388,138 and CRF = 1.3%; Brazil with 17,966,831, 502,586, and CRF = 2.8%; France with 6,168,251, 110,940, and CRF = 1.8%; and Russia with 5,272,328, 127,641, and CRF = 2.4%. Regarding the African continent, the first case was recorded in Egypt on 14 February 2020, with the first cases related to travelers returning from hotspots in Asia, Europe, and the USA. As of 31 May 2021, Africa accounted only for 2.8% and 3.5% of the global COVID-19 infections and deaths, respectively authors analysis of online COVID-19 data [9].

In Africa, the pandemic is exerting great strain on the already fragile economies, health systems, and education systems of all countries affected. Initially confined to major urban areas, the disease is now widespread on the continent. With the continent’s large populations living in high levels of poverty and crowded informal urban settings, coupled with its fragile health systems, there were global fears that the continent would be particularly devasted by the COVID-19 pandemic [10,11,12,13]. However, the COVID-19 pandemic is affecting the African countries differently due to varied economic wealth, health care systems, and governance, which are conditions identified in prior studies to be significant in explaining COVID-19 is spread [14,15,16].

To support evidence-informed policy responses to COVID-19, epidemiological modelling approaches based on both deterministic and stochastic including growth models such as the logistic [17,18,19,20], or the susceptible-infectious-recovered (SIR) mathematical models [19,21] have been used in several countries including Kuwait, China, South Korea, Iran, South Africa, India, and Italy. The models have been applied to estimate short-term prediction of cumulation sizes, spread and transmission processes, and other parameters of the COVID-19 pandemic. The findings could have enabled policymakers to implement suppression measures against the pandemic and to assess their impact on the existing measures. Others use deterministic and mathematical models, and several studies have investigated factors associated with the spread and burden of COVID-19 at ecological levels. For example, these studies have found that highly connected and urbanized areas coincide with a higher COVID-19 burden, compared to their more rural and less connected counterparts [22,23,24]. Socio-demographic and health care resources [22,23,24,25]; environmental factors such as temperatures and air pollution concentration [26]; and migrations from high to low COVID-19 risk areas [27,28] have been shown to associated with COVID-19 burden. The wealthiness of a country or region impacts its COVID-19 testing capacity and, consequently, the number of confirmed and reported COVID-19 cases by the country or region [29].

Several vulnerabilities are available to measure a country’s capacity to detect and respond to epidemic emergencies (for example, the State Party Self-Assessment Annual Reporting (SPAR), which covers topics such as legislation, international health regulations (IHR), coordination, communication, and points of entry). Also, one can use indicators for the Infectious Disease Vulnerability Index (IDVI), which covers topics on demographic, environmental, socioeconomic, and political conditions [10,11,12]. Several of these COVID-19 vulnerabilities exhibit inter-country variations, which may impact the resulting COVID-19 burden and intervention measures.

Using publicly available COVID-19 data, this study sought to examine the spatial relationship between COVID-19 vulnerabilities and the prevalence of COVID-19 at the country-level in the African continent. There are similar studies that have used correlation and regression methods to examine the association between socioeconomic factors and the number of confirmed COVID-19 cases. For example, Cambaza and Viegas [29] studied the correlations between GDP per capita, the number of tests, and the number of confirmed cases of COVID-19 in 13 African countries. Lin et al. [27] used data on the number of COVID-19 cases in the 39 well-developed cities of China and modelled the effects of several socioeconomic indicators. These studies did not account for possible for spatial dependency in the COVID-19 prevalence. However, to understand the impact of neighbouring and the purported influencing factors on COVID-19 prevalence in African countries, spatial statistics analyses could be useful [16]. In other parts of the world, the spatial analysis results have used to assess the impact of neighbouring as well as social-economic factors on the COVID-19 epidemic [22,23,24,25]. These studies analyzed COVID-19 in a specific period. However, regions or countries that may be most vulnerable at later periods may not have been the most affected from the outset [14]. Thus, the effects of some factors on the number of confirmed COVID-19 cases could be period-dependent.

Accordingly, our study was set out to understand how neighbouring countries, as well as socio-economic vulnerabilities, impact COVID-19 prevalence rates of Africa countries using spatial models. We considered two waves of the COVID-19 pandemic, namely from January 2020 to September 2020 (covering the first wave) and from October 2020 to May 2021 (covering the second wave). As Fatima et al. [16] stated, most studies using spatial statistics analyses on COVID-19 have primarily been carried out in China, Brazil, and the USA. To the best of our knowledge, this is the first study to examine how spatial socio-economic vulnerabilities and neighbourliness impact COVID-19 in African countries. An understanding of factors that are influential in explaining country-wide variation in the COVID-19 pandemic in the countries will improve their preparedness to respond against future pandemics. Learning from past infectious pandemics had prepared most of the southeastern Asian countries well. Despite being resource-constrained and having weak health care systems, most of the Southeast Asian countries have unexpectedly attained low COVID-19 infections, partly due to the experience from previous epidemics such as severe acute respiratory syndrome (SARS) and influenza A virus (N1H1) [30,31].

## 2. Methods

### 2.1. Data Sources

Our study covered all sovereign countries of the mainland African continent, except Tanzania whose COVID-19 data were not regularly updated (Somaliland and Western Sahara are disputed areas). Thus, our coverage involved 47 countries. The country-level COVID-19 cases in Africa for the period from February 2020 to May 2021 were extracted from the COVID-19 data repository at Our World (https://ourworldindata.org/coronavirus, accessed on 15 June 2021). We modelled the log of the number of confirmed COVID-19 per 100,000 population in three periods, namely January–September 2020; from October 2020 to May 2021; and the entire period from January 2020 to May 2021. Six country-level COVID-19 vulnerabilities, namely health system; international exposure (travel, trade, tourism or business); population density; age (≥65 years); government transparency; press freedom; and GDP per capita were used in the spatial modelling. These were based on indicators for State Party Self-Assessment Annual Reporting (SPAR), which covers topics such as legislation, international health regulations (IHR), coordination, communication, points of entry, and infectious disease vulnerability index (IDVI), which covers topics regarding demographic, environmental, socioeconomic, and political conditions [10,11,12]. Several of these COVID-19 vulnerabilities exhibit inter-country variations, which may impact the resulting COVID-19 burden and intervention measures. These factors measure a country’s capacity to detect and respond to epidemic emergencies, and they exhibit inter-country variations, which may impact the resulting COVID-19 burden and intervention measures. We also included the number of days since the first confirmed case of COVID-19 for each country. Population density and GDP were taken as continuous while the rest were scaled from 1 to 5, with 5 being the greatest level of COVID-19 vulnerability.

### 2.2. Spatial Regression Models

Anselin [32] and LeSage [33] describe three linear spatial regression models, namely spatial lag, spatial error, and spatial autoregressive condition (SAC) models that one could use to model spatially dependent data. These three were used fit for the regression model of the period COVID-19 prevalence data in our study. We suppose that for each of the countries (N=47), we observe the (logged) COVID-19 prevalence $Y_{i}$. Also, for each country, there is a 1×Q vector $X_{ij}\left(j=1,2,\;\ldots ,Q\right)$ of COVID-19 vulnerabilities, and they are associated with a Q×1 vector β of regression parameters. For the spatial lag, also known as the spatial autoregressive (SAR), model, two more parameters are introduced; one is a N×N spatial weight matrix, W, which quantifies the connection between the countries; and the other parameter is a scale, ρ which measures the strength of spatial dependence in the (logged) COVID-19 prevalence. Now if we let Y be a N×1 vector of logged COVID-19 prevalence data from all the countries, and X be a N×Q matrix of observations of all the countries’ vulnerabilities. Under the normality assumption on Y, the SAR model is then written as,

(1) Y=ρWY+Xβ+ε

where $\varepsilon \sim MVN\left(0,\sigma^{2}I_{N\times N}\right)$. Thus, the SAR model adds a spatially averaged vector as a covariate, reflecting the COVID-19 data from the neighbouring countries to aid in explaining the variation in the prevalence between the countries. The SAR model in (Equation (1)) could also be written as $\left(I_{N\times N}-\rho W\right)Y=X\beta +\varepsilon$ or $Y={\left(I_{N\times N}-\rho W\right)}^{-1}X\beta +{\left(I_{N\times N}-\rho W\right)}^{-1}\varepsilon$. An alternative to the SAR model would be to allow for spatial dependency in the residuals ε. The alternative model, known as the spatial error lag model, is written as

(2) Y=Xβ+υ

where υ is given by

(3) $$\upsilon =\lambda W_{\upsilon }\upsilon +\varepsilon$$

A more general spatial regression model, which combines the SAR and spatial error lag models to contain spatial dependency in both logged prevalence and the residuals, is the spatial autoregressive condition (SAC) model [33]. We fitted all the three spatial regression models, namely SAR, spatial error lag, and SAC to the country-level of COVID-19 period-specific prevalence. A first-order Queen spatial weight matrix, which defines two countries as neighbours when they share a common boundary, was adopted. The results of the spatial regression models were compared to those obtained by fitting an ordinary least squares (OLS) Regression, which would assume that the country-level COVID-19 prevalence rates are independent of each other. The spatial regression analyses were performed in the R package “spdep” developed by Bivand et al. [34].

## 3. Results

Table 1 presents the overall descriptive statistics for the number of cumulative confirmed COVID-19 cases per 100,000 population as of 31 May 2021 as well as selected ecological predictors across the 47 included countries in this study. The average number of days since the first confirmed case in a county was 440.4, with a minimum of 383 days and a maximum of 479 days. As of 31 May 2021, there was a total of 4,748,948 confirmed COVID-19 cases in Africa, with an average, median and range per country of 101,041, 26,963, and 2191 to 1,665,617, respectively. Regarding the confirmed number of COVID-19 related deaths, 2750 were reported per country on average, with a range of 6 to 56,506, which translated into a COVID case fatality rate mean of 2.4. The country average percentage of the population over 65 years old was 3.7. Table 2 shows some of the most and least affected countries in Africa. South Africa was the worst-hit African country at the time of this study, with more than 1.5 million cases and 52,000 deaths (CRF = 3.46%), followed by North African countries such Morocco (500,000 cases, 8700 deaths, CFR = 1.74%); Tunisia (245,000, 8500, CRF = 3.47%); Egypt (194,000, 11,500, CFR = 5.93%), and Algeria (116,000, 3057, CFR = 2.64%). Burundi (2613, 6, CFR = 0.23%) and Liberia (2042, 85, CFR = 4.16%) were some of the least-hit in Africa.

**Figure 1:**
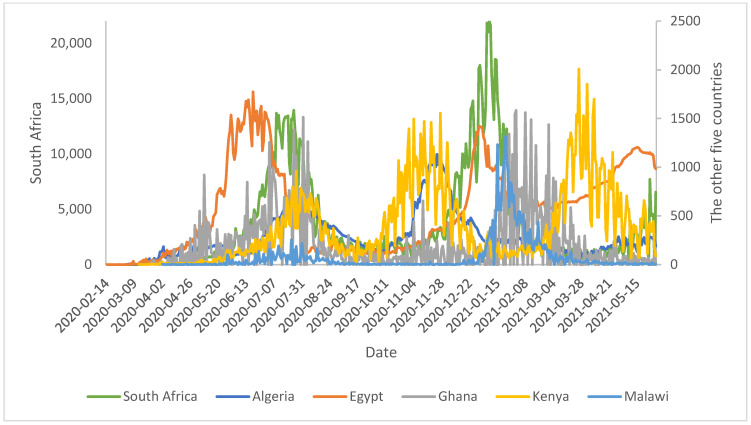
Daily COVID-19 cases by some six countries, as of 31 May 2021.

**Figure 2:**
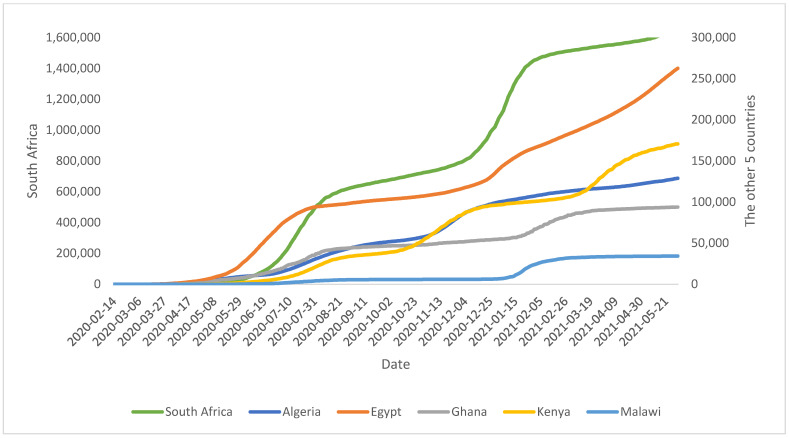
Cumulative number of COVID-19 cases by some six countries, as of 31 May 2021.

The transmission and removal rates of the “rise-fall” of the COVID-19 pandemic trajectories are presented in Figure 1 (for daily numbers of newly infected) and Figure 2 (cumulative confirmed cases) for some countries in Africa, as of 31 May 2021. The trajectories revealed marked differences between the countries, with Kenya having three waves of rise-fall waves of the pandemic while the other five had only two waves. Figure 2 shows that South Africa and Egypt had experienced most of the earlier COVID-19 cases and the two most increases, firstly in the initial phase February to June 2020 and from December 2020 to January 2021.

**Figure 3:**
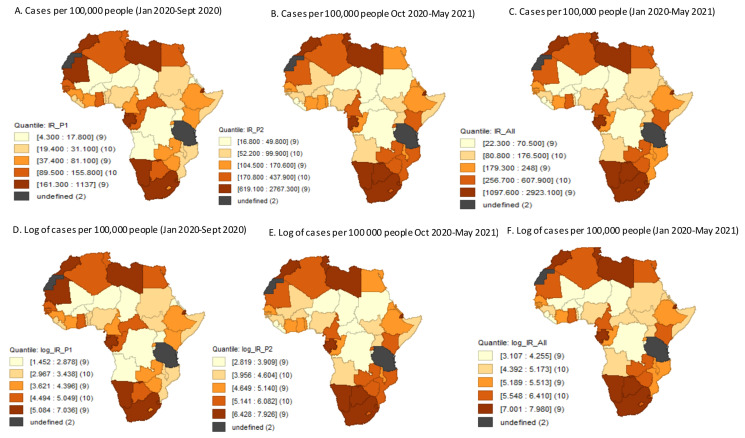
Spatial distributions of confirmed and logged Covid-19 cases per 100,000 people by period, as of 31 May 2021.

**Figure 4:**
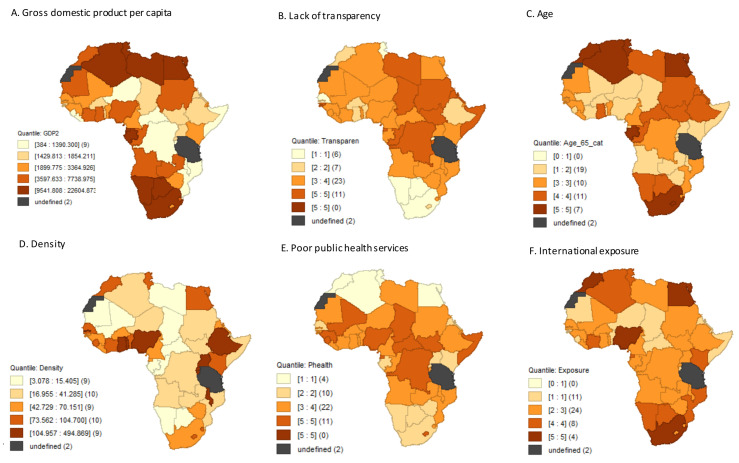
Spatial distribution of the six COVID-19 vulnerabilities as of 31 May 2021.

Figure 3 shows the spatial distribution of confirmed and logged COVID-19 prevalence by period. Countries in the northernmost part of Africa experienced higher burden. The estimates of spatial autocorrelations measured through the global Moran’s I for confirmed and logged COVID-19 prevalence rates were 0.139, 0.334, and 0.339 and 0.252, 0.431, and 0.4 in waves 1, 2 and over the total period, respectively. The positive values of the global Moran’s I suggest that the COVID-19 prevalence in one Africa country may have been related to those in neighbouring countries, especially in wave two. Concerning, COVID-19 vulnerability, countries that showed a higher proportion of older populations and international exposure were the ones that also had higher COVID-19 caseloads. On the other hand, countries that are poorer and less transparent seemed to have lower COVID-19 prevalence (Figure 4).

Firstly, we fitted an ordinary least squares (OLS) regression model to the COVID-19 period prevalence rates and tested the resulting residuals if they were spatially correlated. The OLS residuals in waves one and two had global Moran’s I values of 0.047 and 0.0142 and were non-significant. We, however, further investigated the possibility of spatial lag and spatial error models in the OLS residuals using the Lagrange Multiplier (L-M) test statistics [35]. Both forms of the L-M test were not significant in wave one for either model. Also, there were non-significant L-M test results for the spatial error model in wave two. But, both L-M test forms were significant for the spatial lag model in wave two (*p*-value < 0.05). Thus, a spatial lag model could be preferred for the analysis of COVID-19 prevalence in African countries. Using the Akaike information criterion (AIC), the models that considered the COVID-19 prevalence of neighbouring countries were better fitting models in wave two and for the entire period. The significance of the spatial lag shows that a country’s COVID-19 prevalence was also highly dependent on the COVID-19 of the neighbouring countries. The OLS model was at least better than the spatial models in wave 1 (the initial stage) when COVID-19 cases were only reported in very few countries. Thus, in wave one, COVID-19 cases between countries were very much spatially independent, favoring the OLS model. It was only in wave two, when COVID-19 had a greater geographical diffusion between countries, necessitating the fitting of spatial lag or spatial error models.

Our study included 47 countries and 6 predictors. Thus, we were concerned with the possible multicollinearity between the predictors; the presence of multicollinearity could have affected the stability of the estimates of regression coefficients. A higher degree of multicollinearity can result in the standard errors for the coefficients getting inflated. Only one pair (GDP per capita and International exposure) had Spearman’s correlation coefficients greater than 0.6. The variance inflation factor (VIF) ranged from 1.34 to 2.16, with an average of 1.62, which are far less than 10. Thus, our model results were not affected by any possible multicollinearity.

The results from fitting the OLS and three spatial regression models are summarized in Table 3, Table 4 and Table 5. Countries with relatively high proportions of older populations were associated with a high incidence of confirmed COVID-19 cases while those with lower levels of GDP per capita and poor transparency were associated with reduced COVID-19 burden across all models and waves. An inadequate public health system was only important in the second period where it was related to a reduced incidence of COVID-19 cases.

**Figure A1:**
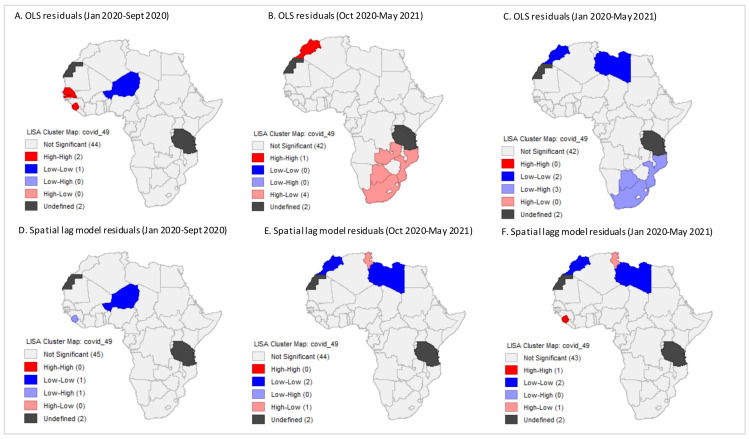
Geographic pattern for residuals of OLS and spatial lag models by period as of 31 May 2021.

Figure A1 in Appendix A show local measures of spatial association of the residuals after fitting the OLS and spatial lag models. For the OLS model, three countries belong to high-high (hot-spot) clusters, having high residuals with similar neighbours and four other countries belong to high-low spatial outliers, having a high value of a residual in the country and with neighbouring countries having low values. The residuals from both models exhibit several low-low spatial clusters where the low value of the residual in a country is associated with neighbouring countries having low value too.

**Figure A2:**
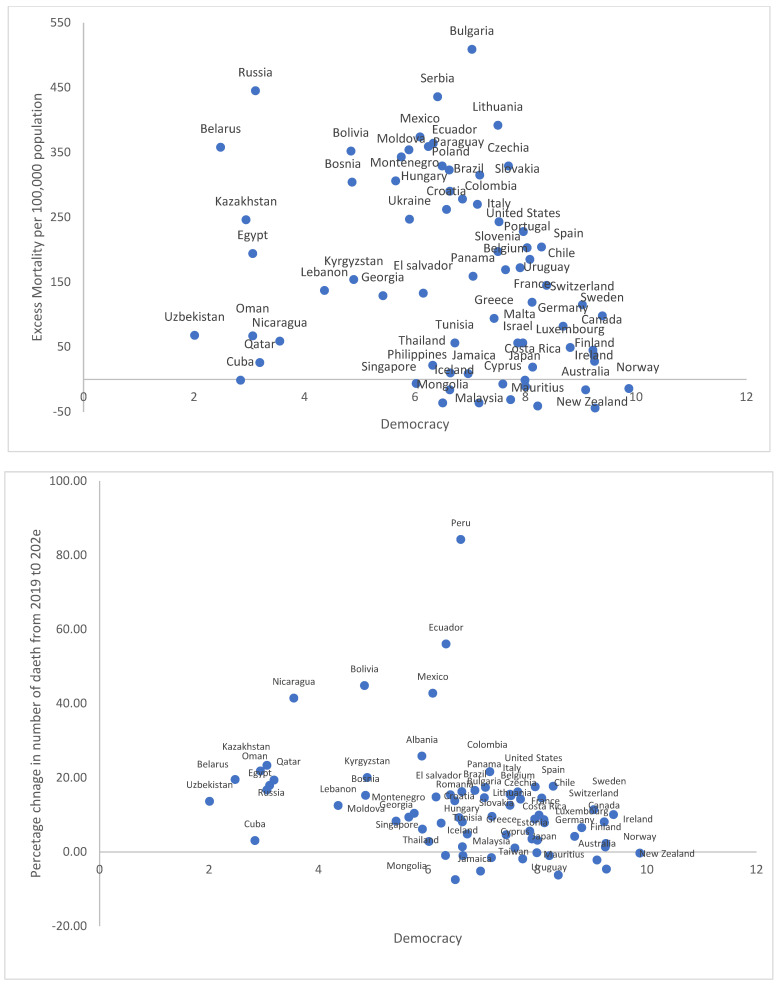
Excess deaths per 100,000 populations and percentage change (2019–2020) in deaths. by EIU democracy index score.

The finding of a negative association between government transparency and the COVID-19 prevalence could be interrogated by comparing the number of deaths in a country before and during COVID-19. However, the challenge for this additional analysis was that most countries in Africa do not record and report annual deaths. Of the 18 out of 54 countries in Africa that record and report annual deaths, only 4 have levels of death registration coverage and cause of death information that meet international standards [36]. Only two countries (Egypt and Tunisia) had reported total deaths from all causes in 2020–2021. So, we analyzed excess mortality data from 73 countries, most of which were non-African, for 2020–2021 (data available at https://github.com/dkobak/excess-mortality as described in Karlinsky and Kobak [37], accessed on 20 September 2021). We also analyzed reported total death for 2019 and 2020 that we extracted from Our World in Data (https://ourworldindata.org, accessed on 20 September 2021). Rather than use transparency in this additional analysis, we used the Democracy Index as measured by the Economist Intelligence Unit (EIU). The Index summarizes ratings on 60 indicators (covering electoral processes and pluralism, civil liberties, the functioning of government, political participation, and political culture) and ranges from 0 to 10, with high values indicative of a high level of democracy [38]. The scatter plots are shown in Figure A2, in Appendix A where both excess death and per cent change (before and after the COVID-19 period) are negatively associated with the democracy score. The correlation between democracy and percentage change in deaths was −0.2581 and excess deaths was −0.3895 (*p*-value < 0.05).

## 4. Discussion

The use of spatial regression models offered insights into why some Africa countries have high or low levels of COVID-19 prevalence. The study found that there was a great level of variability between the countries in Africa concerning COVID-19 prevalence as well as COVID-19 vulnerabilities. GDP per capita, government transparency and the proportion of the population aged 65 years or older were positively associated with the prevalence of COVID-19. Additionally, we found that the COVID-19 prevalence of a country to be highly dependent on those of other neighbouring African countries. The identification of the three influential vulnerabilities affecting the spread of the COVID-19 in the African continent would greatly support efforts aimed at containing the spread of the COVID-19 epidemic. We could not find any other significant associations between COVID-19 and the other vulnerabilities.

Our study findings are consistent with previous research results that found the prevalence of COVID-19 to be positively correlated with wealthiness, transparency and the proportion of the elderly population [22,23,24,25,39,40]. This finding that countries with higher transparency and wealthiness levels are the ones with higher COVID-19 prevalence is surprising and counterintuitive. A possible explanation could be that richer countries can afford to perform more COVID-19 tests, which has a direct positive impact on the number of confirmed cases reported; indeed, this was the conclusion by Cambaza and Viegas [29]. Another explanation is that rich countries tend to be more democratic, and thus more open to more trade and travel, which accelerates the importation as well as the spread of COVID-19 across borders [41], or that they are more transparent in most spheres including data dissemination, which may limit possible data manipulation [38,40]. Yet another explanation is that, paradoxically, richer countries have relatively larger proportions of their population aged 65 years or more, who are more vulnerable to COVID-19 infections [40].

On the other hand, poorer countries are mostly undemocratic, giving them an advantage in dealing with the COVID-19 pandemic since they can forcedly enforce non-pharmaceutical interventions over their populations [38,40]. It could also be that the low COVID-19 prevalence numbers that have been seen in countries with less economic wealth and worse transparency were due to underreporting of the true number of cases due to their insufficient testing capacity. Our further analysis showed that excess deaths during the COVID-19 period were higher in countries with worse democracies (these are generally poorer and less transparent), which may imply that low COVID-19 numbers could be misreporting. The countries could also have been discouraging their people from testing and hiding the reported cases. Prior experience with infectious diseases outbreaks could have facilitated timely and aggressive response in implementing crucial suppressing COVID-19 measures [30,31].

The findings presented in this paper are subject to some limitations. We acknowledge that we could have accounted for the other influencing COVID-19 factors including meteorological variables [41], other socio-economic and health systems variables [24] and population migration and mobility [22,41]. Indeed, the residual maps of spatial error models point to the possibility of ecological predictors that were not controlled for. Since the data used here are based on observations of country-level COVID-19, causality could not be established between predictors and COVID-19 prevalence. The issue of data quality and underreporting of COVID-19 cases could have affected the findings of the associations. Also, the period analyzed here was when COVID-19 vaccinations were not even started in the African continent. Additionally, most governments in Africa had adopted similar preventive and treatment methods. We plan to use the rates of COVID-19 vaccinations and interventions adopted, as these could have changed in recent times.

We are also aware that our results are based on using a country as an administrative division to provide evidence at the country level of planning. Different spatial patterns and interpretations and associations could result if a different aggregation unit could be used. This is a problem of the modifiable areal unit problem (MAUP) in spatial analyses. For example, Wang and Di [26] found that the association between COVID-19 mortality and nitrogen dioxide depended on the aggregated level used, which indicated the presence of MAUP. Thus, we could have assessed and minimize this problem by analysing the data at a lower level (say, regions of the countries) but we were limited by the available data. Changing boundaries of countries to assess changes to the overall spatial patterns and associations was also beyond the scope of the paper.

## 5. Conclusions

This paper employed a series of spatial regression models to assess the impact of neighbouring and socio-economic vulnerability factors on the incidence of COVID-19 in Africa countries. COVID-19 prevalence of the neighbouring countries as well the country’s wealthiness, transparency, and proportion of the population aged 65 or older were found to be influential predictors in explaining disparities in COVID-19 prevalence in African countries. The apparent disadvantage regarding COVID-19 cases among richer and more transparent countries could point to the differences in COVID-19 testing capacity and disease reporting integrities. Even so, our findings will provide countries with evidence to support their responses and interventions to containing the spread of future outbreaks of highly contagious viral infections such as COVID-19. The dependency of a country’s COVID-19 prevalence on those of other neighbouring African countries could reinforce the need for more collaborative COVID-19 non-pharmaceutical interventions and vaccines in reducing the transmission of COVID-19.

## Figures and Tables

**Table 1:** Summary statistics of COVID-19 burden and variables as of 31 May 2021 across 47 African countries.

COVID-19 Burdern Indicator	Mean	SD	Median	Minimum	Maximum
Number of days since 1st case	440.4	13.7	441	383	479
Confirmed number of cases	101,041	254,776	26,963	2191	1,665,617
Confirmed number of cases/100,000	543.8	799.4	211.3	22.3	2923.1
Confirmed number of cases/100,000 *	2.4	0.6	2.3	1.3	3.5
Confirmed number of deaths	2750	8577	517	6	56,506
CFR	2.2	1.5	1.7	0.13	7.4
COVID-19 vulnerability indicator					
Age 65 or older (%)	3.7	1.7	3.1	2.2	10.9
Extreme poverty (%)	34.1	23.6	36.8	0.5	77.6
GDP per capita	5443.8	68	6182.7	2705.4	26,382.3

CFR, case fatality rate; GDP, gross domestic product; SD, standard deviation; * logged.

**Table 2:** The burden of Covid-19 in selected African countries as of 31 May 2021.

Country	Confirmed Cases (No.)	Confirmed Deaths (No.)	CFR (%)
Most affected			
South Africa	1,665,617	56,506	3.39
Morocco	519,216	9147	1.76
Tunisia	345,474	12,654	3.66
Ethiopia	271,541	4165	1.53
Egypt	262,650	15,096	5.75
Least affected			
Burundi	4790	6	0.13
Eritrea	4094	14	0.34
Liberia	2191	86	3.93

**Table 3:** Ordinary least squares (OLS), spatial autoregressive (SAR), spatial error lag, and spatial autoregressive combined (SAC) models for period prevalence (logged): January 2020–September 2020.

COVID-19 Vulnerability	OLS Model		SAR Model		Spatial Error Model		SAC Model	
	Estimate	SE	Estimate	SE	Estimate	SE	Estimate	SE
Constant	1.57	2.72	0.67	3.05	−0.28	2.49	−0.47	2.85
Weak Public Health System	0.06	0.16	0.08	0.15	0.11	0.14	0.13	0.14
High International Exposure	−0.03	0.15	−0.04	0.14	−0.07	0.13	−0.08	0.13
Age 65 or older	0.30	0.12 *	0.30	0.11 *	0.30	0.11 *	0.31	0.12 *
Population Density	−0.0008	0.002	−0.005	0.002	0.00008	0.002	−0.0001	0.002
Low Transparency	−0.25	0.12 *	−0.25	0.11 *	−0.24	0.11	−0.24	0.11 *
Low GDP Capita	−0.0001	0.00003 *	−0.0001	0.00003 *	−0.0001	0.00003 *	−0.0001	0.00003 *
Number of days	0.010	0.01	0.01	0.01	0.02	0.01	0.02	0.01
Spatial lag parameter			0.084				−0.097	
Spatial error parameter					0.207		0.337	
AIC	136.99		138.75		138.5		140.42	

SE, Standard Error; GDP, Gross Domestic Product; AIC, Akaike’s Information Criterion; * *p* < 0.05.

**Table 4:** Ordinary least squares (OLS), spatial autoregressive (SAR), spatial error lag, and spatial autoregressive combined (SAC) models for period prevalence (logged: October 2020–May 2021.

COVID-19 Vulnerability	OLS Model		SAR Model		Spatial Error Model		SAC Model	
	Estimate	SE	Estimate	SE	Estimate	SE	Estimate	SE
Constant	11.38	5.19	4.67	5.10	10.81	4.74	5.22	4.28
Weak Public Health System	−0.30	0.15 *	−0.19	0.13	−0.28	0.13 *	−0.24	0.11 *
High International Exposure	0.04	0.13	0.0005	0.03	0.04	0.12	0.008	0.11
Age 65 or older	0.15	0.11	0.14	0.10	0.15	0.10	0.12	0.08
Population Density	−0.001	0.002	−0.003	0.001	−0.001	0.001	−0.002	0.001
Low Transparency	−0.26	0.11 *	−0.25	0.09 *	−0.26	0.10 *	−0.19	0.08 *
Low GDP Per Capita	−0.0001	0.00003 *	0.00009	0.00003 *	−0.0001	0.00003 *	0.00008	0.00002 *
Number of days	−0.01	0.01	−0.002	0.01	−0.01	0.01	−0.005	0.009
Spatial lag parameter			0.342 *				0.535 *	
Spatial error parameter					0.041		−0.564 *	
AIC	129.25		126.05		131.22		123.42	

SE, Standard Error; GDP, Gross Domestic Product; AIC, Akaike’s Information Criterion; * *p* < 0.05.

**Table 5:** Ordinary least squares (OLS), spatial autoregressive (SAR), spatial error lag, and spatial autoregressive combined (SAC) models for period prevalence (logged): January 2020 to May 2021.

COVID-19 Vulnerability	OLS Model		SAR Model		Spatial Error Model		SAC Model	
	Estimate	SE	Estimate	SE	Estimate	SE	Estimate	SE
Constant	8.83	4.85	3.17	5.11	8.56	4.42	5.22	4.29
Weak Public Health System	−0.20	0.14	−0.11	0.12	−0.19	0.12	−0.18	0.11
High International Exposure	0.002	0.13	0.03	0.11	−0.0003	0.12	−0.006	0.11
Age 65 or older	0.20	0.11	0.18	0.09	0.20	0.10 *	0.17	0.08 *
Population Density	−0.001	0.001	−0.002	0.001	−0.001	0.001	−0.0004	0.001
Low Transparency	−0.26	0.11 *	−0.25	0.09 *	−0.26	0.09	−0.21	0.08
Low GDP Per Capita	−0.001	0.00003 *	−0.0001	0.00003 *	−0.0001	0.00003 *	−0.00008	0.00002 *
Number of days	−0.007	0.01	0.002	0.01	−0.006	0.010 *	−0.004	0.009 *
Spatial lag parameter			0.282				0.448 *	
Spatial error parameter					0.0171		−0.505	
AIC	122.89		121.4		124.89		120.46	

SE, Standard Error; GDP, Gross Domestic Product; AIC, Akaike’s Information Criterion; * *p* < 0.05.

## Data Availability

Our coverage involved 47 countries. The country-level COVID-19 cases in Africa for the period February 2020 to May 2021 were extracted from the COVID-19 data repository at Our World (https://ourworldindata.org/coronavirus accessed on 15 June 2021).

## References

[1] Li Q. (2020). An outbreak of NCIP (2019-nCoV) infection in China—Wuhan, Hubei province, 2019–2020. China CDC Wkly..

[2] Lu H., Stratton C.W., Tang Y.W. (2020). Outbreak of pneumonia of unknown etiology in Wuhan, China: The mystery and the miracle. J. Med. Virol..

[3] Zhu N., Zhang D., Wang W., Li X., Yang B., Song J., Zhao X., Huang B., Shi W., Lu R. (2020). A novel coronavirus from patients with pneumonia in China, 2019. N. Engl. J. Med..

[4] World Health Organization (2020). WHO Director-General’s Opening Remarks at the Media Briefing on COVID-19-11 March 2020.

[5] Wang C., Horby P.W., Hayden F.G., Gao G.F. (2020). A novel coronavirus outbreak of global health concern. Lancet.

[6] Pat A., Adegboye O.A., Adekunle A.I., Rahman K.M., McBryde E.S., Eisen D.P. (2020). Economic Consequences of the COVID-19 Outbreak: The Need for Epidemic Preparedness. Front. Public Health.

[7] Villani L., Pastorino R., Molinari E., Anelli F., Ricciardi W., Graffigna G., Boccia S. (2021). Impact of the COVID-19 pandemic on the psychological well-being of students in an Italian university: A web-based cross-sectional survey. Glob. Health.

[8] Alghamdi A.A. (2021). Impact of the COVID-19 pandemic on the social and educational aspects of Saudi university students’ lives. PLoS ONE.

[9] Ritchie H., Ortiz-Ospina E., Beltekian D., Mathieu E., Hasell J., Macdonald B., Giattino C., Appel C., Rodés-Guirao L., Roser M. (2020). Coronavirus Pandemic (COVID-19). https://ourworldindata.org/coronavirus.

[10] Mehtar S., Preiser W., Lakhe N.A., Bousso A., TamFum J.-J.M., Kallay O., Seydi M., Zumla P.S.A., Nachega J.B. (2020). Limiting the spread of COVID-19 in Africa: One size mitigation strategies do not fit all countries. Lancet Glob. Health.

[11] Gilbert M., Pullano G., Pinotti F., Valdano E., Poletto C., Boëlle P.Y., D’Ortenzio E., Yazdanpanah Y., Eholie S.P., Altmann M. (2020). Preparedness and vulnerability of African countries against importations of COVID-19: A modelling study. Lancet.

[12] Lone S.A., Ahmad A. (2020). COVID-19 pandemic—an African perspective. Emerg. Microbes Infect..

[13] Umviligihozo G., Mupfumi L., Sonela N., Naicker D., Obuku E.A., Koofhethile C., Mogashoa T., Kapaata A., Ombati G., Michelo C.M. (2020). Sub-Saharan Africa preparedness and response to the COVID-19 pandemic: A perspective of early career African scientists. Wellcome Open Res..

[14] Africa Center for Strategic Studies (2020). Mapping Risk Factors for the Spread of COVID-19 in Africa. Africacenter.org/spotlight/mapping-risk-factors-spread-covid-19-africa/.

[15] Sigler T., Mahmuda S., Kimpton A., Loginova J., Wohland P., Charles-Edwards E., Corcoran J. (2021). The socio-spatial determinants of COVID-19 diffusion: The impact of globalisation, settlement characteristics and population. Glob. Health.

[16] Fatima M., O’Keefe K.J., Wei W., Arshad S., Gruebner O. (2021). Geospatial Analysis of COVID-19: A Scoping Review. Int. J. Environ. Res. Public Health.

[17] Shen C.Y. (2020). Logistic growth modelling of COVID-19 proliferation in China and its international implications. Int. J. Infect. Dis..

[18] Roosa K., Lee Y., Luo R., Kirpich A., Rothenberg R., Hyman J., Yan P., Chowell G. (2020). Real-time forecasts of the COVID-19 epidemic in China from February 5th to February 24th, 2020. Infect. Dis. Model..

[19] Almeshal A.M., Almazrouee A.I., Alenizi M.R., Alhajeri S.N. (2020). Forecasting the Spread of COVID-19 in Kuwait Using Compartmental and Logistic Regression Models. Appl. Sci..

[20] Reddy T., Shkedy Z., Janse van Rensburg C., Mwambi H., Debba P., Zuma K., Manda S. (2021). Short-term real-time prediction of total number of reported COVID-19 cases and deaths in South Africa: A data driven approach. BMC Med. Res. Methodol..

[21] Cooper I., Mondal A., Antonopoulos C.G. (2020). A SIR model assumption for the spread of COVID-19 in different communities. Chaos Solitons Fractals.

[22] Sun F., Matthews S.A., Yang T.C., Hu M.H. (2020). A spatial analysis of COVID-19 period prevalence in US counties through June 28, 2020: Where geography matters?. Ann. Epidemiol..

[23] Cuadros D.F., Xiao Y., Mukandavire Z., Correa-Agudelo E., Hernández A., Kim H., MacKinnon N.J. (2020). Spatiotemporal transmission dynamics of the COVID-19 pandemic and its impact on critical healthcare capacity. Health Place.

[24] Raymundo C.E., Oliveira M.C., Eleuterio T.D.A., André S.R., da Silva M.G., Queiroz E.R.D.S., Medronho R.D.A. (2021). Spatial analysis of COVID-19 incidence and the sociodemographic context in Brazil. PLoS ONE.

[25] Saffary T., Adegboye O.A., Gayawan E., Elfaki F., Kuddus M.A., Saffary R. (2020). Analysis of COVID-19 Cases’ Spatial Dependence in US Counties Reveals Health Inequalities. Front. Public Health.

[26] Wang Y., Di Q. (2020). Modifiable areal unit problem and environmental factors of COVID-19 outbreak. Sci. Total Environ..

[27] Lin Y., Zhong P., Chen T. (2020). Association Between Socioeconomic Factors and the COVID-19 Outbreak in the 39 Well-Developed Cities of China. Front. Public Health.

[28] Nyasulu J.C.Y., Munthali R.J., Nyondo-Mipando A.L., Pandya H., Nyirenda L., Nyasulu P.S., Manda S. (2021). COVID-19 pandemic in Malawi: Did public sociopolitical events gatherings contribute to its first-wave local transmission?. Int. J. Infect. Dis..

[29] Cambaza E.M., Viegas G.C. (2021). COVID-19: Correlation between gross domestic product, number of tests, and confirmed cases in 13 African countries. J. Public Health Epidemiol..

[30] Nit B., Samy A.L., Tan S.L., Vory S., Lim Y., Nugraha R.R., Lin X., Ahmadi A., Lucero-Prisno D.E. (2021). Understanding the Slow COVID-19 Trajectory of Cambodia. Public Health Pract..

[31] Nguyen Thi Yen C., Hermoso C., Laguilles E.M., De Castro L.E., Camposano S.M., Jalmasco N., Cua K.A., Isa M.A., Akpan E.F., Ly T.P. (2021). Vietnam’s success story against COVID-19. Public Health Pract..

[32] Anselin L. (1988). Spatial Econometrics: Methods and Models.

[33] LeSage J.P. An Introduction to Spatial Econometrics, Revue D’économie Industrielle [Online], 123 | 3e Trimestre 2008, Document 4, Online since 15 September 2010, Connection on 19 April 2019. http://journals.openedition.org/rei/3887.

[34] Bivand R.S., Pebesma E., Gomez-Rubio V. (2013). Applied Spatial Data Analysis with R.

[35] Anselin L., Bera A.K., Florax R., Yoon M.J. (1996). Simple Diagnostic Tests for Spatial Dependence. Reg. Sci. Urban Econ..

[36] Sankoh O., Dickson K.E., Faniran S., Lahai J.I., Forna F., Liyosi E., Kamara M.K., Jabbi S.B., Johnny A.B., Conteh-Khali N. (2020). Births and deaths must be registered in Africa. Lancet Glob. Health.

[37] Karlinsky A., Kobak D. (2021). Tracking excess mortality across countries during the COVID-19 pandemic with the World Mortality Dataset. eLife.

[38] Kapoor M., Malani A., Ravi S., Agrawal A. (2020). Authoritarian governments appear to manipulate COVID data. arXiv.

[39] Liang L.-L., Tseng C.-H., Ho H.J., Wu C.-Y. (2020). Covid-19 mortality is negatively associated with test number and government effectiveness. Sci. Rep..

[40] Annaka S. (2021). Political regime, data transparency, and COVID-19 death cases. SSM Popul. Health.

[41] Wang Q., Dong W., Yang K., Ren Z., Huang D., Zhang P., Wang J. (2021). Temporal, and spatial analysis of COVID-19 transmission in China and its influencing factors. Int. J. Infect. Dis..

